# UMIAD-EGMF: unsupervised medical image anomaly detection based on edge guidance and multi-scale flow fusion

**DOI:** 10.1186/s42492-026-00215-3

**Published:** 2026-03-02

**Authors:** Zhirong Li, Guangfeng Lin, Dou Zhang, Rongxin Huang, Jing Yang

**Affiliations:** 1 Information Science Department, Xi’an University of Technology, Xi’an, 710048 Shaanxi Province China; 2 State Key Laboratory of Public Big Data, Guizhou University, Guiyang, 550025 Guizhou Province China

**Keywords:** Medical image analysis, Anomaly detection, Unsupervised learning, Edge guidance, Multi-scale flow fusion

## Abstract

Medical imaging technology has advanced rapidly in recent years; however, abnormalities in medical images are often rare and complex, making sample labels difficult to obtain for supervised learning of detection models. Existing unsupervised anomaly detection methods, which are the mainstream approaches, often struggle with issues such as blurred edges and varying scales of abnormal regions. To address these issues, a novel unsupervised method for medical image anomaly detection is proposed: unsupervised medical image anomaly detection based on edge guidance and multi-scale flow fusion (UMIAD-EGMF). This method excavates rich edge information with scale adaptation and progressively identifies discriminative information for anomaly detection. UMIAD-EGMF captures contextual information around anomaly boundaries via low-level feature fusion (enhancing boundary details with the edge guidance module; EGM), integrates EGM-extracted edge information into deeper features using the edge aggregation module, and merges multi-scale feature maps to capture common anomaly features (subtle and significant) through multi-scale flow fusion. Experiments on breast ultrasound images (BUSI), brain magnetic resonance imaging (brain MRI), and head computed tomography (head CT) datasets demonstrate that UMIAD-EGMF outperforms the state-of-the-art methods. Specifically, on the BUSI dataset, the segmentation area under the precision-recall curve for object localization (AUPRO) of UMIAD-EGMF reaches 63.36%, surpassing that of the multi-scale low-level feature enhancement U-Net (MLFEU-net) by 0.01%; on the brain MRI dataset, its segmentation AUPRO is 90.83%, outperforming that of MLFEU-net by 0.33%; and on the head CT dataset, its segmentation AUPRO is 62.24%, exceeding that of MedMAE by 2.37%.

## Introduction

**Fig. 1:**
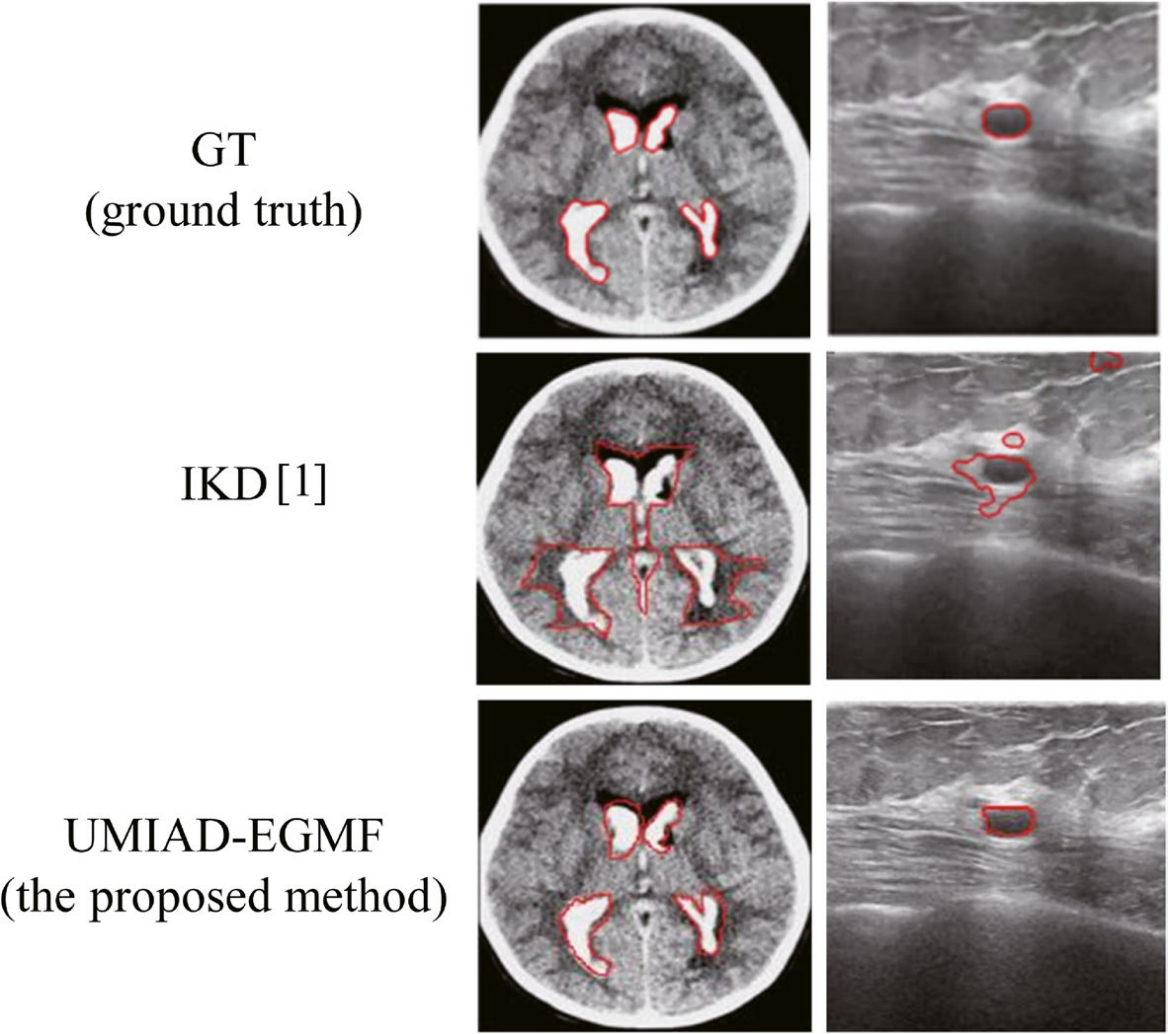
Influence of the intricately blurred edge information and the multi-scale nature of abnormal regions (compare with informative knowledge distillation (IKD) [1]). The left column shows the different detection results of the multi-scale anomaly region and the right column shows the different detection results of the complex edges of the anomaly region. UMIAD-EGMF: Unsupervised medical image anomaly detection based on edge guidance and multi-scale flow fusion

In recent years, medical imaging technology has advanced significantly owing to deep learning. The traditional manual diagnosis of medical images is often influenced by a doctor’s medical experience, knowledge structure, physiological status (for example, visual fatigue), and other subjective factors. In contrast, deep learning-based methods for medical image anomaly detection can significantly reduce the influence of these human factors on the diagnosis. These methods can help doctors identify abnormalities faster and more accurately, thereby improving the diagnostic efficiency and accuracy. However, owing to the complexity of disease manifestations, unsupervised model learning and the discrimination of abnormal regions face major challenges in the detection of medical image anomalies. Specific issues include intricate blurred edge information and multi-scale characteristics of abnormal regions, as shown in Fig. 1. Intricately blurred edge information is prevalent in the early stages of some diseases and is exacerbated by shadows and scattered noise, particularly in ultrasound images. This makes it difficult for the model to accurately delineate the boundaries of abnormal regions. Multi-scale characteristics refer to the presence of abnormal regions of varying sizes in medical images, which complicate the effective processing these size differences in existing network models.

Existing methods [2, 3] attempt to mitigate edge blur by increasing the width and depth of the network or by modifying the concatenation and skip connection operations, which enable each layer to fully extract image features and boundary details. The high-level feature fusion and recalibration U-Net [4] was derived from the U-Net architecture by modifying skip pathways using local feature reconstruction and a feature fusion mechanism that represents detailed contextual information in high-level features. Lee et al. [5] proposed the multi-receptive field (MRF) module for feature aggregation and feature fusion. The MRF module utilizes the adaptive selection mechanism of multi-branch convolution kernels to achieve adaptive feature extraction. The MRF module fuses the input feature maps using the convolution kernels of different receptive fields, making the obtained information more comprehensive. Shao et al. [6] introduced an artificial eeural network (ANN) based on edge detection methods for edge information processing. To adapt to multi-scale abnormal regions, a deep convolutional neural network (CNN) is used to discriminate between normal and abnormal tissues using a Gaussian pyramid representation for multi-scale analysis [7], a multi-scale context-guided deep network captures both global and local contexts to guide model training [8], and a multi-scale visual deformation model employs a position-coded transform model and a local CNN to extract global and local information [9]. Another approach [10] proposed a novel multi-scale parallel-branch architecture called multi-scale parallel focal self-attention U-Net (MP-FocalUNet). On the encoder side of MP-FocalUNet, dual-scale sub-networks are used to extract information from different scales. Dai et al. [11] proposed a multi-scale multi-task model framework called MTF-UNet. It does not use different-sized convolution kernels for multi-scale feature extraction but instead uses same-sized kernels with varying numbers of convolutions to obtain multi-scale multi-level features.

However, these methods often suffer from limitations in their ability to generalize to different types of medical image datasets because of blurred edges and varying scales of abnormal regions. To address these issues, unsupervised medical image anomaly detection based on edge guidance and multi-scale flow fusion (UMIAD-EGMF) is proposed to mine the common edge information of image regions to accurately detect anomalies in different medical images. In UMIAD-EGMF, the edge guidance module (EGM) serves as a boundary prior for segmentation by extracting shallow image edge features, the edge aggregation module (EAM) enhances segmentation performance by aggregating feature information, and multi-scale flow fusion (MFF) captures common anomalous features with subtle and significant multi-dimensional information through the base distribution.

The contributions of this study are as follows:

(1) The contextual information of the boundaries of image regions can describe edge detail information from the EGM to the EAM through a hierarchical progression strategy to enhance the anomaly detection performance. The EGM can merge low-level appearance features via interactive attention weight learning, and the EAM can then employ attention mechanisms and residual connections to aggregate low- and high-level features.

(2) Multi-scale adaptation detection can eliminate the differences between various scales of image regions to fit anomalous areas using MFF. As different scales of base distributions can theoretically approximate any image region, MFF can exchange local and global information within multi-scale feature pyramids to enhance the detection and segmentation of abnormal regions.

(3) The promising results in medical anomaly detection (MAD) demonstrate that the proposed UMIAD-EGMF outperforms the existing state-of-the-art (SOTA) methods on different types of medical image datasets. Furthermore, the proposed UMIAD-EGMF achieves better performance in different edge information extraction and edge aggregation methods.

To address the blurred edges and varying scales of abnormal regions, existing methods typically utilize edge detection methods and generative-based approaches for edge information mining and region information reconstruction. These studies are reviewed in the following subsections.

### Edge detection methods

There are numerous edge-based detection algorithms for medical images, each employing different strategies to identify regions with significant gray level changes, thereby effectively segmenting abnormal areas. Some of these methods utilize classical edge detection techniques, such as Sobel, Laplacian of Gaussian (LoG), and Canny operators. The Sobel operator detects edges by calculating the gradient of grayscale variations in the image. The LoG operator combines Gaussian smoothing with second-order derivatives to detect edges efficiently. The Canny edge detector accurately identifies edges through a multi-step process that includes Gaussian smoothing, gradient calculation, non-maximum suppression, and hysteresis thresholding. Although traditional methods are typically computationally simple, they are susceptible to noise and often struggle to accurately identify complex edges and details.

Modern deep learning algorithms effectively address the limitations of traditional edge detection methods. For example, Shao et al. [6] used a Canny operator-based edge detector as the training output for an ANN. Compared to conventional operators, such as Sobel and Canny, the neural network-based edge detection method captures more comprehensive edge information in magnetic resonance imaging (MRI) medical images and processes it almost three times faster than traditional methods. Several proposed methods based on U-Net [2, 3, 12] can extract richer edge features. A novel CNN architecture [2] integrates an inception-res module and a densely connected convolutional module into a U-Net framework. The connectivity and skip connections are modified to incorporate an EGM that integrates features from all layers [3]. The boundary-aware context neural network [13] follows the classic encoder-decoder structure, which embeds a pyramid edge feature extraction module, mini-multi-task learning module, interactive attention layer, and cross-feature fusion module for object segmentation. The cross-level feature aggregation network [12] introduced a boundary prediction network to generate boundary-conscious features, enhance hierarchical features, and produce finer segmentation maps. The multi-scale low-level feature enhancement U-Net (MLFEU-net) [14] has been presented as a multi-scale low-level feature enhancement U-Net consisting of a U-Net structure, a low-level feature enhancement block, and a parallel multi-scale feature fusion module. Specifically, the low-level feature enhancement block enhances detailed information during shallow downsampling and further feature selection. Meanwhile, in the neck of the U-Net, the parallel multi-scale feature fusion module uses dilated convolutions of different scales to provide receptive fields of varying sizes and efficiently merges filtered shallow and deep features. However, the parallel multi-scale feature fusion module relies on fixed dilation rates and cannot dynamically adapt to extreme lesion scales or establish edge-semantic consistency when fusing shallow and deep features. Recently, MedViTV2 [15] proposed a novel hybrid architecture that integrates kolmogorov-arnold network (KAN) layers into transformers for the first time. MedViTV2 introduces an efficient KAN block that reduces the computational load while improving the accuracy of the original MedViT [16]. To counteract the fragility of MedViT when scaled up, MedViTV2 also uses an enhanced dilated neighborhood attention mechanism. This is an adaptation of the efficient fused dot-product attention kernel, which can capture global context and expanding receptive fields. This allows the model to be scaled effectively and addresses the issue of feature collapse. Furthermore, a hierarchical hybrid strategy is introduced to efficiently stack local feature perception and global feature perception blocks and balance local and global feature perceptions to enhance performance. However, MedViTV2 is primarily designed for image-level classification and lacks explicit optimization for pixel-level anomaly segmentation. It does not incorporate edge guidance to handle blurred lesion boundaries or MFF to capture subtle anomalies (for example, micro-hemorrhages in head computed tomography (CT) scans). This makes it less suitable for clinical anomaly detection tasks that require the precise delineation of lesions.

In summary, existing methods, including U-Net variants and hybrid transformer-KAN models, such as MedViTV2, neglect the hierarchical, progressive interaction of edge information at different scales when describing the commonalities of different types of medical images. Most classification-oriented hybrid models fail to establish effective connections between the edge details and semantic features, resulting in poor performance in pixel-level anomaly localization. The proposed UMIAD-EGMF model attempts to hierarchically model the interaction of low-level appearance features via the EGM while progressively integrating high-level semantic features via the EAM, with the aim of improving anomaly detection and segmentation accuracy.

### Generative-based approaches

Generative-based approaches include two types of detection and segmentation methods: generative adversarial network (GAN)-based and flow model methods.

GAN-based methods can be used to reconstruct the regional information from medical images. The anomaly GAN (AnoGAN) [17] first proposed the application of GANs for anomaly detection by mitigating their inherent instability through a feature matching mechanism. However, AnoGAN suffers from slow training and has a limited capability in reconstructing complex images. Unlike previous patch-based approaches, the anomaly variational autoencoder GAN (AnoVAEGAN) [18] addresses these limitations by employing a deep spatial autoencoder to capture the comprehensive global features of normal images, thereby enhancing the realism of the reconstructed samples. The fast AnoGAN [19] leverages the wasserstein-GAN [20] framework to significantly improve training speed while capturing smoother representations. MADGAN [21], an unsupervised multi-stage anomaly detection method, was the first method for reconstructing multiple adjacent brain MRI slices using a GAN network to detect anomalies in multi-sequence MRI images. Building on these advancements, the memory-augmented multi-level cross-attentional masked autoencoder [22] integrated a transformer-based approach with masked autoencoders for accurate anomaly detection.

Flow-based methods not only explicitly learn data distributions but also leverage their reversibility to transform data without information loss and achieve high-quality data reconstruction and generation. For example, FastFlow [23] locates features in normal regions of an input image at the center of the distribution, whereas features in abnormal regions deviate significantly from the center. Similarly, DifferNet [24] implemented a normalizing flow for image-based anomaly detection to handle variations in the defect size via multi-scale feature maps. The conditional normalizing flow framework adopted for anomaly detection [25] constructs a multi-scale feature pyramid by incorporating feature maps with varying efficiency levels and receptive fields. In addition, conditional normalizing flow framework adopted for anomaly detection extends the normalization process to pixel-by-pixel anomaly localization by estimating the likelihood of the feature vector at each location. However, it treats each feature vector independently, ignoring the spatial context information. This lack of contextual awareness impairs global perception, resulting in incoherent localization outcomes. To address this issue, the multi-scale flow-based framework for unsupervised anomaly detection (MSFlow) [26] first extracts feature maps from the initial three stages, down samples them via average pooling, and then feeds them into a normalizing flow. The extracted multi-scale feature maps are then fed into their respective asymmetric parallel flows, with information from different scales and perceptual fields exchanged through a fusion flow. Finally, multi-scale likelihood maps are aggregated using various strategies tailored to different anomaly detection and localization tasks. In addition, medical masked autoencoder (MedMAE) [27] optimizes self-supervised learning for medical images but lacks explicit distribution modeling of flows.

Most current unsupervised anomaly detection algorithms based on flow models are primarily used for industrial defect detection. Drawing inspiration from MSFlow, this study extends the fusion flow model to unsupervised medical image anomaly detection. The proposed UMIAD-EGMF effectively handles lesions of varying and unpredictable sizes in medical images by incorporating edge information mining to enhance the detection and segmentation of anomalous regions. Furthermore, existing hybrid architectures, including transformer-based models such as MedViTV2 and flow-based models such as CFlow, suffer from feature isolation. CFlow uses canny edges as auxiliary inputs but does not fuse them with flow-based semantic features. MedViTV2 focuses on balancing global and local features for classification without linking edge details to segmentation tasks. This leads to inconsistent edge and anomaly localizations. In contrast, the EGM-EAM-MFF pipeline of UMIAD-EGMF realizes progressive integration: the EGM extracts context-aware edges, the EAM embeds these edges into the semantic space of the flow, and MFF ensures that the edge information propagates across flow scales. This end-to-end integration avoids the two-stream separation problem of existing hybrids, filling the gap between classification-oriented hybrid models and the need for clinical anomaly segmentation.

## Methods

### Network architecture of UMIAD-EGMF

**Fig. 2:**
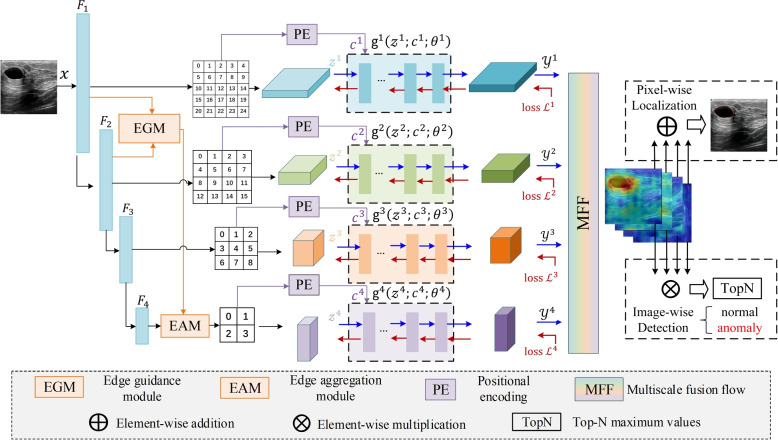
Network architecture of the proposed UMIAD-EGMF model. $c^k$ refers to the condition vector generated through position encoding, $z^k$ represents the feature vector obtained through multi-scale pyramid pooling operation, $g^k (z^k;c^k;\theta ^k)$ is the *k*-th independent decoder model, $\theta ^k$ represents the parameter of the decoder, $y^k$ indicates the output of the *k*-th independent decoder model, and $L^k$ refers to the loss of the *k*-th independent decoder model

The proposed model employs a codec architecture. The encoder was designed as a CNN feature extractor with multi-scale pyramidal pooling. Initially, four layers of multi-scale features, $F_1, F_2, F_3,$ and $F_4$, are extracted from the input image using this encoder. The EGM extracts edge information from the extracted features. The shallow features $F_1$ and $F_2$ interact with the different scale features for edge information extraction via the EGM, and the edge information can be incorporated with the high-level feature $F_4$ through the EAM. Subsequently, the feature vector $z^k$ from the multi-scale pyramid pooling operation and the conditional vector $c^k$ from the position encoding are fed into $k$ independent decoders $g^k(z^k;c^k;\theta ^k)$ for the multi-scale flow mapping process. This process integrates multi-scale normalized streams and a multi-scale stream fusion module to enhance their information exchange. In the final anomaly detection stage, the model uses an additive aggregation strategy for pixel-level anomaly localization and a multiplicative aggregation strategy for image-level anomaly detection. The anomaly score of the image is determined by calculating the average of the top $N$ scores in the image-level anomaly score map. Figure 2 shows the network architecture of the proposed UMIAD-EGMF.

### EGM

**Fig. 3:**
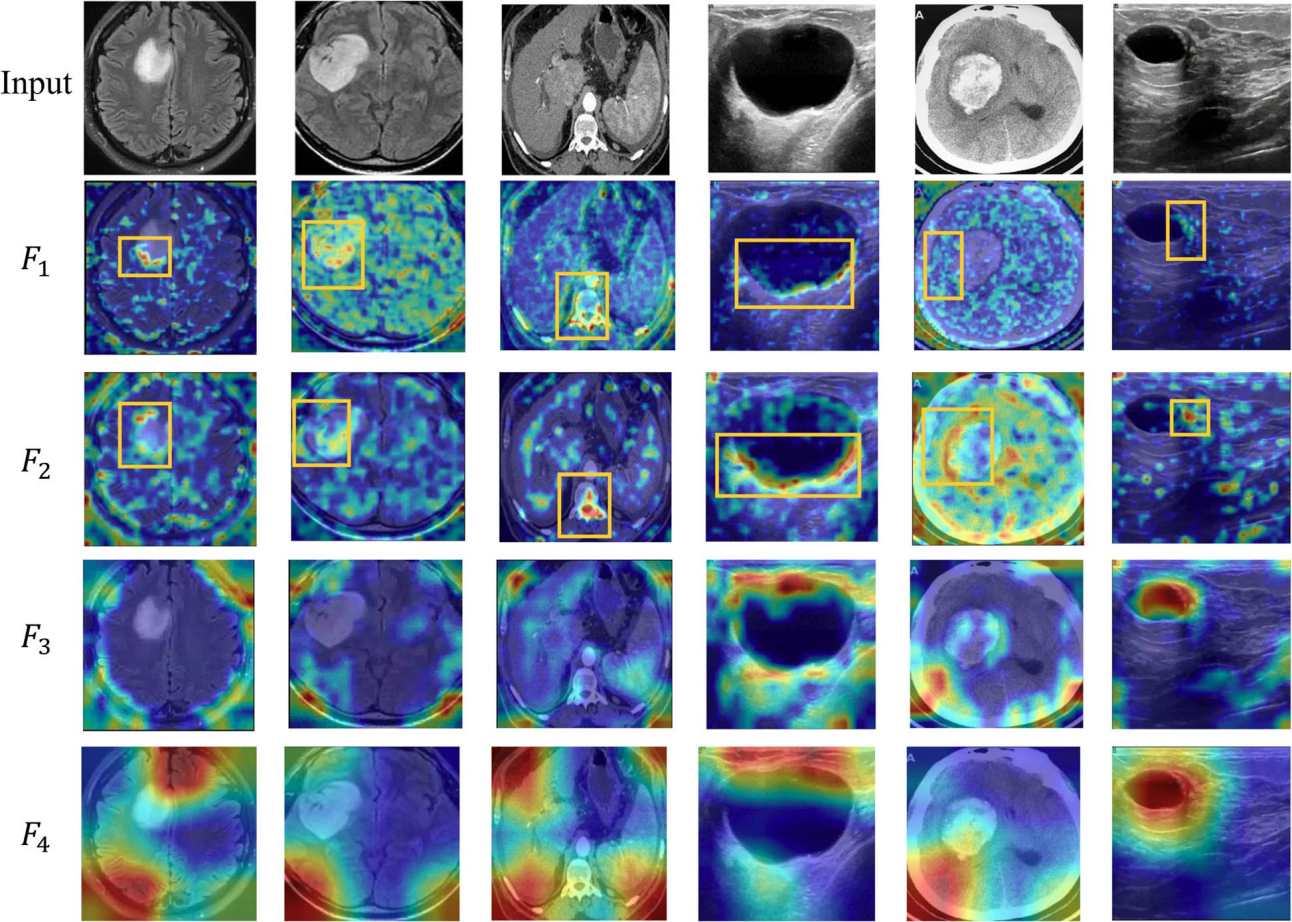
Heat map of different layer features $F_1, F_2, F_3 ,F_4$ extracted from the ResNet18 backbone network in the various column images

**Fig. 4:**
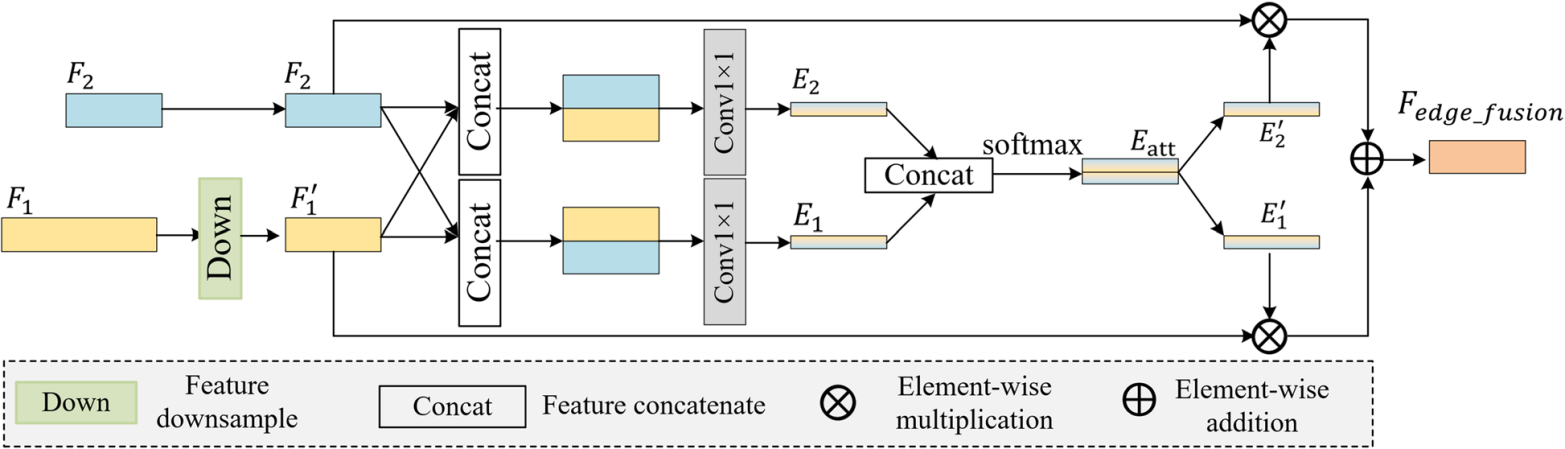
Schematic of the edge guidance module. $F_1$ and $F_2$ are the low-level features

This subsection introduces the EGM for extracting edge information from lesion regions in medical images. The proposed UMIAD-EGMF uses ResNet18 [28] as the backbone network. ResNet18 can generate different layers with four features:$F_1, F_2, F_3$, and $F_4$. Figure 3 shows the heat maps of these features at different layers in the various images. The low-layer features ($F_1$ and $F_2$) primarily capture local information, such as edges and textures, whereas the high-layer features ($F_3$ and $F_4$) focus on all semantic information of the image. To enhance edge information extraction, the EGM can not only fuse these features ($F_1$ and $F_2$) from the first two layers but also mine the edge information to improve the detection accuracy of abnormal regions. Figure 4 illustrates a schematic of the EGM.

Specifically, the EGM first downsamples the shallow feature $F_1$, which captures fine textures, such as small lesion edges, to match the size of $F_2$, which captures coarser local structures. The downsamples feature of $F_1$ is denoted as $F_1'$. To ensure that the edge detection covers both fine and coarse details, the key to blurring ultrasound lesion images, two sets of initial edge attention weights ($E_1$ and $E_2$) are generated by swapping the order of $F_1'$ and $F_2$ during concatenation as follows:

1 $$\begin{aligned} E_1 = \text {Conv}^{1}_{1 \times 1} \left(\text {Concat}\left(F_1', F_2\right)\right) \end{aligned}$$

2 $$\begin{aligned} E_2 = \text {Conv}^{2}_{1 \times 1} (\text {Concat}(F_2, F_1') \end{aligned}$$

Equation 1 prioritizes $F_1'$, the downsampled fine texture, by placing it first in the concatenation, making $E_1$ sensitive to small edge details, such as the boundary of a 2 mm breast cyst. Equation 2 prioritizes $F_2$, the coarse structure, by reversing the order, making $E_2$ focus on larger edge contours, such as the outer edge of a breast tumor. Different gated convolutions $\text {Conv}_{1 \times 1}$ focus on different parts of the feature map by learning the weights for each position in the input feature map, allowing the model to adaptively fuse information from different feature maps. After convolution, the features are processed through cascading operations and softmax processing as follows:

3 $$\begin{aligned} E_{\text {att}} = \text {softmax}(\text {Concat}(E_1, E_2))\end{aligned}$$

where $E_{\text {att}}$, which balances the attention between small edge details and large edge contours, is the interaction weight between different weight distributions. After normalization using the softmax activation function, the interaction weight is between 0–1. Furthermore, the normalized attention vector is split into $E_1'$, the refined attention weight for small edge details, and $E_2'$, the refined attention weight for large edge contours, for different low-layer feature fusions.

4 $$\begin{aligned} E_1', E_2' = \text {split}(E_{\text {att}}) \end{aligned}$$

5 $$\begin{aligned} F_{\text {\textit{edge\_fusion}}} = E_1' \otimes F_1' \oplus E_2' \otimes F_2 \end{aligned}$$

where $\text {split}(\cdot )$ denotes the split operation, $\otimes$ represents pixel-wise multiplication after dimension broadcasting, and $\oplus$ represents pixel-wise addition. $F_{\text {\textit{edge\_fusion}}}$, the feature that integrates small details and large contours, denotes the edge enhancement fusion. This fusion enables the model to focus on important parts of the feature map, thereby improving the edge information for medical image anomaly detection.

Taking a breast ultrasound images dataset (BUSI) of benign lesion image (as shown in Visualization subsection) as an example, the lesion edge is blurred by ultrasound speckle noise, making it difficult for traditional methods to distinguish the lesion from the surrounding fat tissue. The EGM fuses low-level features $F_1$ (texture details) and $F_2$ (local edges) via interaction attention (Eq. 5), which assigns higher attention weights to pixel regions with gray-level changes (lesion boundaries), while suppressing noise regions. This results in $F_{\text {\textit{edge\_fusion}}}$. The EAM further aggregates this edge information with a high-level feature $F_4$, correcting the mis-localization of CFlow [25] which marks three small false-positive regions and reduces the false-positive rate.

### EAM

The EGM effectively extracts edge information. However, aggregating this information and high-level semantic information within the network remains a challenge because of noise and redundancy. To optimize the use of edge-aware features in resolving these issues, the EAM is introduced to further extract information regarding edge fusion and capture features that are aggregated at different scales. The incorporation of edge information in conjunction with the channel attention mechanism facilitates the capacity of the model to discern anomalous patterns in unlabeled data during unsupervised learning, particularly in regions where the edges are imperceptible. Consequently, the edge fusion feature $F_{\text {\textit{edge\_fusion}}}$ is fed from the EGM into the channel attention block (CAB) [29] to obtain the edge enhancement feature $F_{\text {\textit{edge\_fusion}}}^{\text {\textit{att}}}$. It optimizes the importance of different feature channels, assigning higher weights to channels containing lesion edges and lower weights to channels dominated by noise, as follows:

6 $$\begin{aligned} F_{\text {\textit{edge\_fusion}}}^{\text {\textit{att}}} = \text {CAB} (F_{\textit{edge\_fusion}}) \end{aligned}$$

where $F_{\text {\textit{edge\_fusion}}}^{\text {\textit{att}}}$ is the edge feature enhanced by the channel attention mechanism and CAB ($\cdot$) is the channel attention operation. The enhanced edge feature $F_{\text {\textit{edge\_fusion}}}^{\text {\textit{att}}}$ is then merged with the deeper feature $F_4$ in the network and processed through a 3 $\times$ 3 convolution to obtain the feature $F_{\text {\textit{edge}}}^{\text {\textit{cat}}}$. The feature contains both specific lesion edge details and global lesion attributes.

7 $$\begin{aligned} F_{\text {\textit{edge}}}^{\text {\textit{cat}}} = \text {Conv}_{3 \times 3} \left(\text {concat}\left(F_{\text {\textit{edge\_fusion}}}^{\text {\textit{att}}}, F_4\right)\right) \end{aligned}$$

**Fig. 5:**
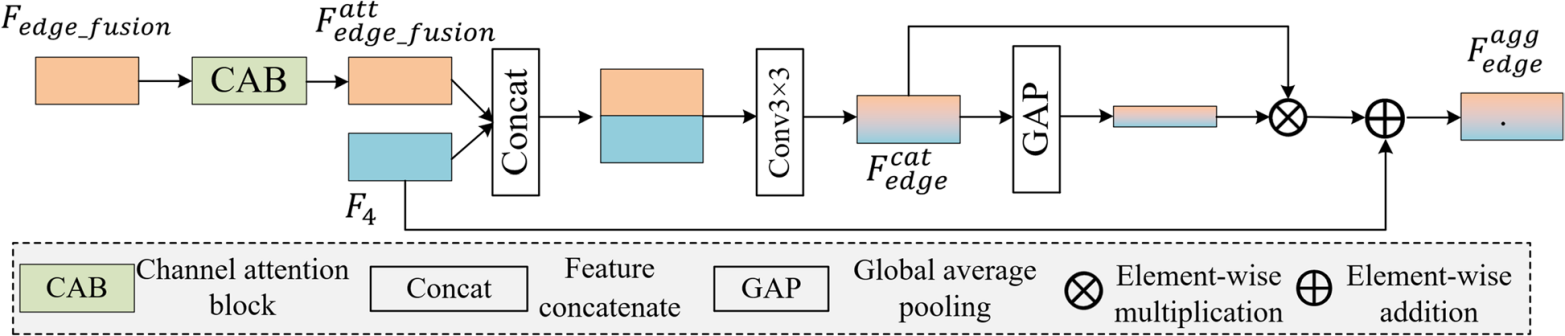
Schematic of the EAM.$F_4$ is the high-level feature,$F_{\text {\textit{edge\_fusion}}}$ refers to the edge fusion feature extracted by the EGM, $F_{\text {\textit{edge\_fusion}}}^{\text {\textit{att}}}$ represents the edge enhancement feature obtained by $F_{\text {\textit{edge\_fusion}}}$ through the CAB, $F_{\text {\textit{edge}}}^{\text {\textit{cat}}}$ indicates the combination of $F_4$ and $F_{\text {\textit{edge\_fusion}}}^{\text {\textit{att}}}$, and $F_{\text {\textit{edge}}}^{\text {\textit{agg}}}$ refers to the final aggregated feature

The ability of the model to capture edge details is enhanced by fusing information from different feature layers in the spatial dimension. Feature $F_{\text {\textit{edge}}}^{\text {\textit{cat}}}$ is then fed into the global average pooling (GAP) layer. The output of the GAP layer enhances the cascaded features, improving the perception of edge information by the model, and resulting in an aggregated feature $F_{\text {\textit{edge}}}^{\text {\textit{agg}}}$. This is similar to an enhanced version of the lesion edge map that preserves the subtle edge details of the lesion while integrating its overall semantic information.

8 $$\begin{aligned} F_{\text {\textit{edge}}}^{\text {\textit{agg}}} = \text {GAP} (F_{\text {\textit{edge}}}^{\text {\textit{cat}}}) \otimes F_{\text {\textit{edge}}}^{\text {\textit{cat}}} \oplus F_4 \end{aligned}$$

where GAP ($\cdot$) represents the GAP operation. In addition, to preserve high-layer feature information, residual connections can be used to fuse high-level features in the current hierarchy, thereby enhancing the expressiveness and learning efficiency of the network. A schematic of the EAM is presented in Fig. 5.

### MFF module

**Fig. 6:**
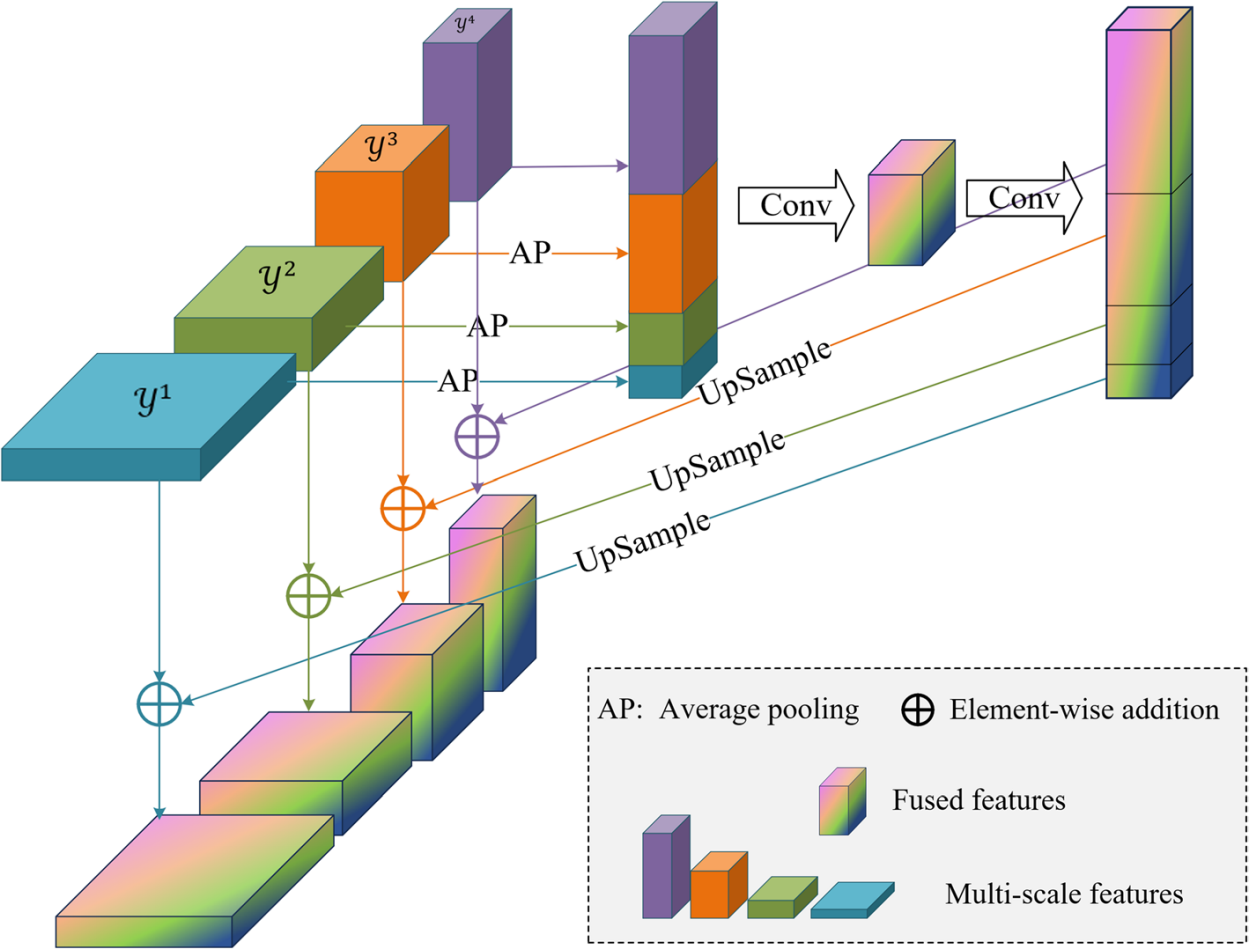
Schematic of the multi-scale flow fusion module. $y^k$ ($k=1,2,3,4$) indicates the output of the *k*-th independent decoder model for mapping flow features

Medical images typically contain complex biological tissue structures exhibiting different details and features at various scales. The effective acquisition and fusion of these multi-scale features are crucial for improving the accuracy of medical image anomaly detection. Existing methods utilize multi-scale strategies for operating on features at each scale in isolation and fail to fully exploit multi-scale interactions. To address this issue, this study introduces an MFF module that captures anomalous features ranging from subtle to significant by pooling and averaging feature maps at different scales. This module provides rich and multi-dimensional information to the model, thereby significantly enhancing its accuracy and sensitivity in identifying and localizing anomalous structures of various sizes. A schematic of the MFF module is shown in Fig. 6.

First, an average pooling operation is performed on the multi-scale features $y^1, y^2,$ and $y^3$, retaining the fine details and supplementing them with deep semantics, from the first three levels to match the dimensions of $y^4$, retaining the global semantics and supplementing them with shallow details. The pooled features $y_{\text {AP}}^1, y_{\text {AP}}^2,$ and $y_{\text {AP}}^3$ are then concatenated with $y^4$, combining the feature information from different levels and enabling the model to comprehensively sense the image structure at various scales. Next, the concatenated features are fused using a 3 $\times$ 3 convolution operation to better capture the correlations between the features. Finally, the first three levels of features are upsampled to restore their original sizes, and the original features are added to the fused features. This process retains information from the original features to further improve the ability of the model to characterize the features.

9 $$\begin{aligned} \left\{y_{\text {f}}^1, y_{\text {f}}^2, y_{\text {f}}^3, y_{\text {f}}^4\right\}= f_{\text {f}} \left(\text {AP}\left(y^1\right),\mathrm{AP}\left(y^2\right),\mathrm{AP}\left(y^3\right), y^4\right) \end{aligned}$$

where $y_{\text {f}}^k$ denotes the *k*-th layer features of the MFF module, $f_{\text {f}}$ represents the MFF module, and $\text {AP}(\cdot )$ denotes the average pooling operation. $y^k$ ($k=1,2,3,4$) are the features after multi-scale normalizing flow decoding as follows:

10 $$\begin{aligned} y^k = \text {g}^k \left( z^k; c^k;\theta ^k \right) \end{aligned}$$

where $c^k$ denotes the condition vector generated through position encoding, $z^k$ denotes the feature vector obtained through the multi-scale pyramid pooling operation, and $\theta ^k$ denotes the decoder parameter. $g^k (z^k;c^k;\theta ^k)$ converts the distribution of normal tissue features into a standard distribution, making it easier to identify lesion features that deviate from this distribution. It is the *k*-th independent decoder from the normalizing flow [30], which can transform the probability density through a series of reversible and differentiable mappings. In this manner, different feature levels in an image can be captured, thereby locating abnormal areas at different scales in medical images. In the proposed UMIAD-EGMF, multiple affine coupling blocks can be stacked to construct sufficiently complex distributions. $g^k (z^k;c^k;\theta ^k)$ is composed of eight affine coupling blocks $h_n(z^k;c^k;\theta ^k)$($n=1,2,3,4,5,6,7,8$) which handle the irregular feature distribution of blurred ultrasound lesions. The affine coupling block proposed by ref. [25] can be expressed as follows:

11 $$\begin{aligned} g^k(z^k;c^k;\theta ^k)= h_1(z^k;c^k;\theta ^k)\circ h_2(z^k;c^k;\theta ^k)\circ \cdots \circ h_8(z^k;c^k;\theta ^k) \end{aligned}$$

where $\circ$ denotes the cascading operation.

UMIAD-EGMF uses the maximum likelihood of the targets for training, which is equivalent to minimizing the loss $\mathcal {L}^k$ in the *k*-th layer. According to CFlow [25], loss $\mathcal {L}^k$ can be reformulated as follows:

12 $$\begin{aligned} \mathcal {L}^k \approx D_{KL} \left[ p_{Z} (\boldsymbol{z}^k) \Vert \hat{p}_{Z} (\boldsymbol{z}^k| \boldsymbol{c}^k, \boldsymbol{\theta }^k) \right] \frac{1}{N} \sum \limits _{i=1}^{N} \left[ \frac{\Vert \boldsymbol{u}_i^k\Vert _2^2 }{2} - \log \left| \det \boldsymbol{J}_i^k \right| \right] + \text {const} \end{aligned}$$

where $p_{Z} (\boldsymbol{z}^k)$ represents the true probability density of the feature $\boldsymbol{z}^k$ in *Z* feature space, and $\hat{p}_{Z} (\boldsymbol{z}^k| \boldsymbol{c}^k, \boldsymbol{\theta }^k)$ represents the prediction probability density of the feature $\boldsymbol{z}^k$ under the condition of $\boldsymbol{c}^k$ and $\boldsymbol{\theta }^k$ in *Z* feature space. The random variable $\boldsymbol{u}_i^k= g^{-1}(z_i^k, c_i^k, \theta ^k)$ is Jacobian $J_i^k = \nabla _{z^k} g^{-1}(z_i^k, c_i^k, \theta ^k)$.

### Computational complexity

This subsection analyzes and explains the computational complexity of the proposed UMIAD-EGMF, which is mainly divided into four parts: EGM, EAM, normalizing flow, and MFF. In these parts, it is assumed that the maximum number of input channels, output channels, and height and width of features in convolutional operations are $C_{in}$, $C_{out}$, *H*, and *W* respectively, and that the maximum dimension of the features in multiplicative operations is *N*.

First, the computational complexity of the EGM is mainly borne by two $1 \times 1$ convolution kernels and two multiplication operations. It is expressed as $O(2 \times C_{in}\times C_{out} \times H \times W+ 2 \times N^3)$.

Second, the computational complexity of the EAM involves the CAB module with a maximum feature dimension *K*, a $3 \times 3$ convolution kernel, and a multiplication operation. It is expressed as $O( K^3 + 3 \times 3 \times C_{in}\times C_{out} \times H \times W + N^3 )$.

Third, the eight decoupling blocks contribute to the computational complexity of the normalizing flow decoder module. Each block involves two sets of double $3 \times 3$ convolution kernels and multiplication operations. Therefore, the computational complexity of this module is $O((2 \times 3 \times 3 \times C_{in}\times C_{out} \times H \times W + N^2)\times 2 \times 8)$.

Finally, the MFF module mainly includes two $3 \times 3$ convolution kernels. Thus, the computational complexity of the MFF module is $O(2 \times 3 \times 3 \times C_{in}\times C_{out} \times H \times W )$.

Therefore, the total computational complexity of the proposed UMIAD-EGMF is as follows:

13 $$\begin{aligned} & O ((2 \times C_{in}\times C_{out} \times H \times W+ 2 \times N^3)+ (K^3 + 3 \times 3 \times C_{in}\times C_{out} \times H \times W + N^3)+((2 \times 3 \times 3 \times C_{in}\times C_{out} \times H \times W + N^2)\times 2 \times 8)+ (2 \times 3 \times 3 \times C_{in}\times C_{out} \times H \times W) ) \nonumber \\ & = O(K^3+317\times C_{in}\times C_{out} \times H \times W+ 16\times N^2+ 3\times N^3) \end{aligned}$$

## Results

### Datasets

In unsupervised learning, only normal images are required as input during training. Medical image datasets included normal and abnormal images and lesion region mask data for abnormal images; however, these data are often scarce in public medical datasets. Therefore, in addition to validating the model on the public BUSI dataset [31], this also obtained brain MRI [32] and head CT datasets [33] to validate the model. All three datasets are publicly available and cover different modalities (ultrasound, MRI, and CT), common diseases (breast lesions, gliomas, and brain hemorrhages), and clinical acquisition protocols. Their diversity validates the generalization of UMIAD-EGMF across real-world medical imaging scenarios.

**Fig. 7:**
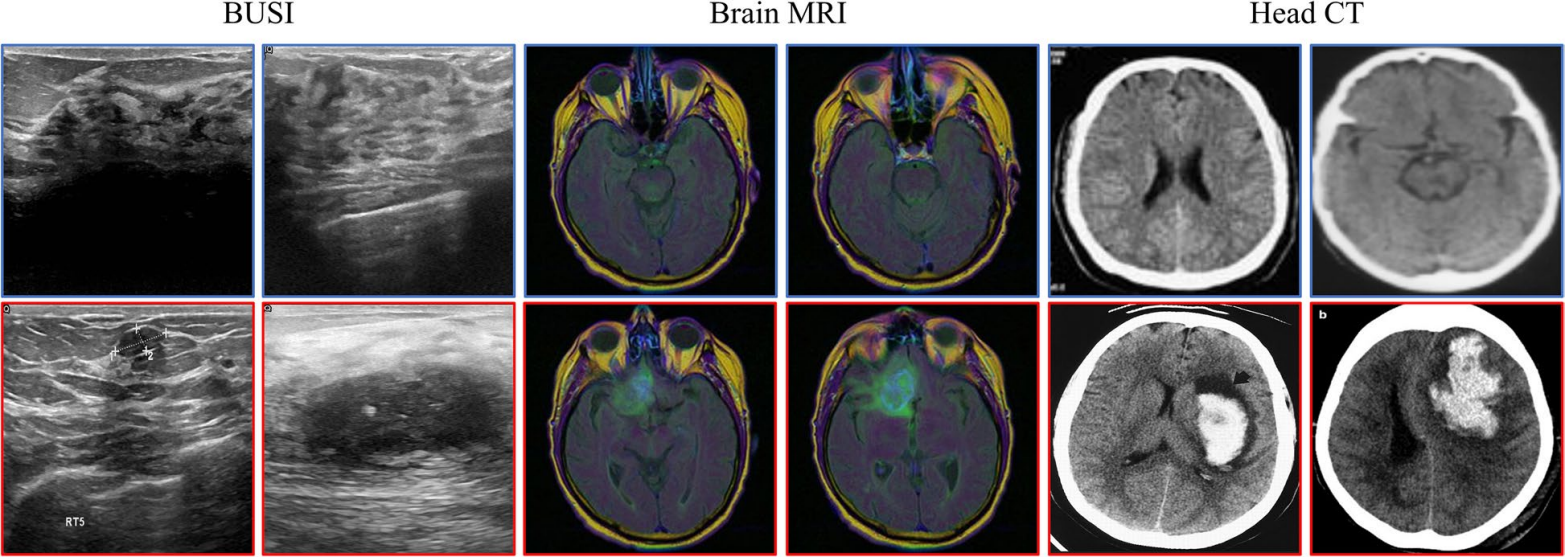
Example of a medical anomaly detection dataset. The first row shows examples of normal samples (framed in blue), while the second row shows examples of abnormal samples (framed in red). The first two columns are from the breast ultrasound images dataset (BUSI), the following four columns are from the brain magnetic resonance imaging (MRI) dataset, and the last six columns are from the head computed tomography (CT) dataset

The BUSI dataset contained 746 breast ultrasound images from 600 patients aged 25–75 years, with an average image size of 500 $\times$ 500 pixels. This dataset included 437 benign, 210 malignant, and 99 normal images. Healthy images in the brain MRI dataset were obtained from the national institutes of health human connectome project and collected from more than 1,200 healthy volunteers. Each volunteer provided four types of images: structural brain images (T1w and T2w), resting-state functional MRI, and task-state functional MRI. Diseased images were sourced from the brain tumor segmentation dataset [32], which contained the MRI scans of 351 subjects, with each sample including T1, T1ce, T2, and FLAIR MRI images. For this experiment, the brain MRI dataset comprised of 88 normal and 154 diseased images. The head CT dataset included CT slices of the normal brain and hemorrhage images. Ninety normal and 100 diseased images were used in this experiment. The statistics of the datasets are listed in Table 1. Examples of the three datasets are shown in Fig. 7.

**Table 1:** Statistics of the medical anomaly detection datasets

Dataset	Train	Test (normal)	Test (anomaly)
Breast ultrasound images dataset	99	34	647
Brain magnetic resonance imaging dataset	88	10	154
Head computed tomography dataset	90	10	100
Total	277	54	901

### Evaluation metrics

MAD involves abnormality image classification (image level) and abnormal region segmentation (pixel level). Therefore, this study used both image- and pixel-level evaluation metrics, including image- and pixel-level area under the receiver operating characteristic curve (AUROC), area under the precision-recall curve for object localization (AUPRO), recall, and precision. The image-level AUROC assesses the binary classification performance of the model at the image-level, whereas the pixel-level AUROC evaluates the ability of the model to localize lesion regions in medical images. The pixel-level AUROC tends to favor larger abnormalities, whereas the AUPRO metric ensures that both large and small abnormalities are equally important for localization. Pixel-level recall represents the ratio of truly abnormal pixels, such as lesion pixels, that are accurately identified, which is the key to covering small or scattered lesions. Pixel-level precision indicates the ratio of pixels predicted as abnormal to those that are truly abnormal, ensuring accurate lesion boundary localization without mislabeling adjacent normal tissues. Additionally, recall and precision typically exhibit a trade-off, where improving one metric may compromise the other, and their balance should be adjusted based on specific clinical tasks.

### Implementation details

Taking an image with an input size of 256 $\times$ 256 as an example, Table 2 lists the main configuration parameters of the network structure, including the input and output sizes of the different network layers. The network contained three parts: the feature extraction phase, multi-scale stream decoding phase, and fusion flow phase. In the feature extraction phase, ResNet18 was used as the backbone network and initialized with pre-trained weights from the ImageNet dataset. The training parameters were as follows: the number of training iterations was 100, the Adam optimizer was used with a learning rate of 0.0002, batch size was 32, and a normalizing flow model was generated using the FrEIA library [34].

**Table 2:** Network details parameter sheet (Input batch size: 32 for all layers)

Network layer	Input size	Output size	Key parameter
**Feature extractor**			
Input_size	(32, 3, 256, 256)	(32, 3, 256, 256)	
Conv2d + BN + ReLu	(32, 3, 256, 256)	(32, 64, 64, 64)	Output_channels = 64, kernel_size = 7 × 7
Maxpool	(32, 64, 64, 64)	(32, 64, 32, 32)	Output_channels = 64, kernel_size = 3 × 3
Layer1	(32, 64, 32, 32)	(32, 64, 32, 32)	Output_channels = 64, kernel_size = 3 × 3
Layer2	(32, 64, 32, 32)	(32, 128, 16, 16)	Output_channels = 128, kernel_size = 3 × 3
Layer3	(32, 128, 16, 16)	(32, 256, 8, 8)	Output_channels = 256, kernel_size = 3 × 3
Layer4	(32, 256, 8, 8)	(32, 512, 4, 4)	Output_channels = 512, kernel_size = 3 × 3
**Multi_flow**			
Decoder layer1	(32, 64, 32, 32)	(32, 64, 32, 32)	
Decoder layer2	(32, 128, 16, 16)	(32, 128, 16, 16)	
Decoder layer3	(32, 256, 8, 8)	(32, 256, 8, 8)	
Decoder layer4	(32, 512, 4, 4)	(32, 512, 4, 4)	
**Fusion_flow**			
AvgPool2d layer1	(32, 64, 32, 32)	(32, 64, 4, 4)	Kernel_size = 3, stride = 8, padding = 0
AvgPool2d layer2	(32, 128, 16, 16)	(32, 128, 4, 4)	Kernel_size = 3, stride = 4, padding = 0
AvgPool2d layer3	(32, 256, 8, 8)	(32, 256, 4, 4)	Kernel_siz e= 3, stride = 2, padding = 0
Layer4	(32, 512, 4, 4)	(32, 512, 4, 4)	
	(32, 64, 4, 4)		
Concat	(32, 128, 4, 4)	(32, 960, 4, 4)	
	(32, 256, 4, 4)		
	(32, 512, 4, 4)		
Conv2d	(32, 960, 4, 4)	(32, 240, 4, 4)	Output_channels = 240, kernel_size = 1 × 1
Conv2d	(32, 240, 4, 4)	(32, 960, 4, 4)	Output_channels = 960, kernel_size = 1 × 1
		(32, 64, 4, 4)	
Split	(32, 960, 4, 4)	(32, 128, 4, 4)	
		(32, 256, 4, 4)	
		(32, 512, 4, 4)	
UpSample layer1	(32, 64, 4, 4)	(32, 64, 32, 32)	
UpSample layer2	(32, 128, 4, 4)	(32, 128, 16, 16)	
UpSample layer3	(32, 256, 4, 4)	(32, 256, 8, 8)	
Layer4	(32, 512, 4, 4)	(32, 512, 4, 4)	

### Comparison with the SOTA methods

This subsection compares the proposed UMIAD-EGMF with nine methods (supplemented with MedViTV2), including CFlow [25], u-shaped normalizing flow (UFlow) [35], MSFlow [26], attention-augmented differentiable top-k feature adaptation (ADFA) [36], informative knowledge distillation (IKD) [1], reverse distillation for anomaly detection (RD4AD) [37], MedMAE [27], MLFEU-net [14], and MedViTV2 [15]. These methods fall into three categories: (1) CFlow, UFlow, and MSFlow, which are based on the normalizing flow framework; (2) ADFA, IKD, and RD4AD, which rely on the knowledge distillation paradigm; and (3) MedMAE, MLFEU-net, and MedViTV2, where MedMAE is based on VAE, MLFEU-net is based on U-Net, and MedViTV2 is a transformer-based method. Specifically, CFlow treats features at each scale independently but ignores the correlations between different scales. UFlow incorporates interactive features via a u-shaped network but loses detailed information owing to different sampling strategies. Although MSFlow uses an asymmetric parallel flow architecture and multi-scale information fusion to enhance multi-scale perception, it neglects the importance of edge information for segmenting abnormal regions and fails to adequately represent refined features. ADFA is an unsupervised medical image anomaly detection method that improves the separability of normal and abnormal medical image features through feature adaptation. However, this method is only effective for images with high heterogeneity and is unsuitable for detecting blurred normal and abnormal images with high homogeneity. Both IKD and RD4AD utilize knowledge distillation frameworks to discriminate cross-domain data but still require training support from cross-domain knowledge. To address the above limitations of existing methods, the proposed UMIAD-EGMF integrates edge information into a multi-scale flow model through an unsupervised learning approach, enabling accurate detection of anomalous images and regions. All comparative experiments used images of size 256 $\times$ 256.

**Table 3:** Performance (%) comparison of the proposed UMIAD-EGMF with the SOTA methods on breast ultrasound images, brain magnetic resonance imaging, and head computed tomography datasets

Method	DET.AUROC	Seg.AUROC	Seg.AUPRO	Recall	Precision
BUSI dataset					
CFlow (2022)[25]	92.43	74.76	59.16	75.82	82.35
UFlow (2024)[35]	65.84	72.81	32.73	70.15	78.62
MSFlow (2024)[26]	85.89	76.78	62.19	77.53	84.18
ADFA (2023)[36]	95.40	73.20	60.47	78.21	85.21
IKD (2022)[1]	94.63	63.19	44.60	72.34	81.79
RD4AD (2022)[37]	87.20	68.00	51.30	73.88	82.65
MedMAE (2025)[27]	93.85	74.62	61.53	77.54	82.12
MLFEU-net (2025)[14]	94.92	**79.21**	63.35	**79.34**	83.53
MedViTV2 (2025)[15]	94.70	76.50	61.90	77.80	83.20
UMIAD-EGMF	**95.83**	77.81	**63.36**	78.91	**86.79**
Brain MRI dataset					
CFlow (2022)[25]	87.18	84.84	55.48	82.45	85.73
UFlow (2024)[35]	79.68	70.62	40.31	73.28	80.19
MSFlow (2024)[26]	85.89	78.78	58.19	80.67	83.92
ADFA (2023)[36]	**94.60**	79.40	55.31	**85.24**	86.87
IKD (2022)[1]	89.22	83.32	50.54	83.15	84.62
RD4AD (2022)[37]	90.70	82.70	57.50	82.83	85.19
MedMAE (2025)[27]	88.91	81.77	57.30	83.21	85.45
MLFEU-net (2025)[14]	90.50	**87.50**	59.10	84.30	84.10
MedViTV2 (2025)[15]	90.20	83.80	57.80	83.50	84.80
UMIAD-EGMF	90.83	86.34	**59.40**	84.67	**87.52**
Head CT dataset					
CFlow (2022)[25]	80.70	84.77	57.49	81.69	83.58
UFlow (2024)[35]	63.50	71.40	31.36	71.85	79.43
MSFlow (2024)[26]	73.70	80.99	46.36	79.82	82.76
ADFA (2023)[36]	**85.70**	83.20	60.48	84.19	**84.35**
IKD (2022)[1]	83.10	87.37	61.31	**85.47**	83.91
RD4AD (2022)[37]	74.50	87.40	61.80	85.22	83.68
MedMAE (2025)[27]	80.70	82.42	59.87	82.56	81.23
MLFEU-net (2025)[14]	82.50	**87.60**	**62.65**	83.56	82.30
MedViTV2 (2025)[15]	82.20	84.20	60.90	82.90	82.70
UMIAD-EGMF	82.90	86.76	62.24	83.98	83.12

DET.AUROC denotes the AUROC of detection at the image-level. Seg.AUROC and Seg.AUPRO represent the AUROC and AUPRO of segmentation at the pixel-level, respectively. **Bold** and underlined values indicate the best and the second-best performance

Table 3 lists the experimental results of the proposed UMIAD-EGMF compared with those of the eight methods across the three datasets. The results demonstrate that UMIAD-EGMF achieved superior performance in most evaluation metrics, with particular advantages in pixel-level anomaly localization, which is consistent with its design goal of multi-scale anomaly detection. A detailed analysis of the results obtained using the datasets is presented as follows.

On the BUSI dataset, UMIAD-EGMF outperformed all methods for DET.AUROC (95.83%) and achieved the highest Seg.AUPRO (63.36%). For DET.AUROC, it exceeded ADFA (the second-ranked method) by 0.43% and surpassed MedViTV2 (94.70%) by 1.13%, demonstrating stronger image-level anomaly identification. For pixel-level segmentation, its Seg.AUPRO (63.36%) was 1.46% higher than MedViTV2 (61.90%), and its precision (86.79%, the highest) effectively balanced missed detections and false alarms. This advantage stems from the multi-scale pooling and edge fusion module of UMIAD-EGMF, which accurately analyzes the boundaries of breast lesions. This is crucial for distinguishing small/irregular ultrasound abnormalities, whereas MedViTV2 lacks this ability because of its classification-oriented design.

For the brain MRI dataset, UMIAD-EGMF balanced image-level detection and pixel-level segmentation, maintaining the leading segmentation performance. Although its DET.AUROC (90.83%) was 3.77% lower than that of ADFA (94.60%), it outperformed MedViTV2 (90.20%) by 0.63% for DET.AUROC and achieved a 1.60% higher Seg.AUPRO (59.40% *vs* 57.80% for MedViTV2). The DET.AUROC advantage of ADFA comes from its top-k operator and pre-trained network generalization, whereas the multi-scale decoder and flow fusion of UMIAD-EGMF enhance local anomaly perception, making it more effective for fine-grained brain lesion localization. Its highest precision (87.52%) further reduced false positives in normal brain tissue, which is challenging for MedViTV2 owing to its lack of edge-semantic consistency.

On the head CT dataset, UMIAD-EGMF delivered competitive segmentation performance. Its Seg.AUPRO (62.24%) ranked second only to MLFEU-net (62.65%) and surpassed MedViTV2 (60.90%) by 1.34%. Its recall (83.98%) and precision (83.12%) were among the top three metrics, ensuring reliable localization. While IKD (87.37%) and RD4AD (87.40%) had slightly higher Seg.AUROC via distillation-based supervisory signals, UMIAD-EGMF outperformed MedViTV2 in all pixel-level metrics, confirming its adaptability to high-density tissues and complex anatomical structures in Head CT. This advantage stems from the EGM and MFF of UMIAD-EGMF, which enhance the perception of lesion edges by the model.

Table 4 lists the inference time and number of model parameters of the proposed UMIAD-EGMF and SOTA methods on the BUSI dataset. As the input image size was uniformly set to 256 $\times$ 256, selecting the BUSI dataset for this experiment yielded representative results, where consistent input conditions ensured the comparability of the inference time and parameter metrics across the methods.

UMIAD-EGMF achieved an inference time of 55.2 ms, which falls within the real-time threshold for clinical medical imaging (less than 100 ms) and thus satisfies the real-time requirements of MAD. The inference time of UMIAD-EGMF was only 2.4 ms longer than that of MedMAE (52.8 ms); this increment is negligible for most clinical scenarios and is accompanied by significant improvements in detection performance. The inference time of UMIAD-EGMF was 1.8 ms shorter than that of MedViTV2, which indicates that the proposed method is superior to current transformer-based methods in terms of detection speed and accuracy. In terms of model parameters, UMIAD-EGMF maintained a moderate parameter count compared to SOTA methods, avoiding excessive computational overheads that could hinder deployment on resource-constrained clinical devices. This confirms the feasibility of UMIAD-EGMF for real-time clinical use and clarifies the rational trade-off between model complexity, inference efficiency, and detection performance.

**Table 4:** Inference time per image and number of model parameters comparison of different methods on the breast ultrasound images dataset

Method	Inference time per image (ms)	Number of model parameters
CFlow (2022) [25]	**42.3**	13 M
MSFlow(2024) [26]	51.7	15 M
ADFA(2023) [36]	47.5	19 M
MLFEU-net(2025) [14]	54.7	**12.3 M**
MedMAE(2025) [27]	52.8	40 M
MedViTV2(2025) [15]	57.0	29.6 M
UMIAD-EGMF	55.2	14 M

**Bold** values indicate the best model performance (the smallest inference time or the least number of model parameters)

**Fig. 8:**
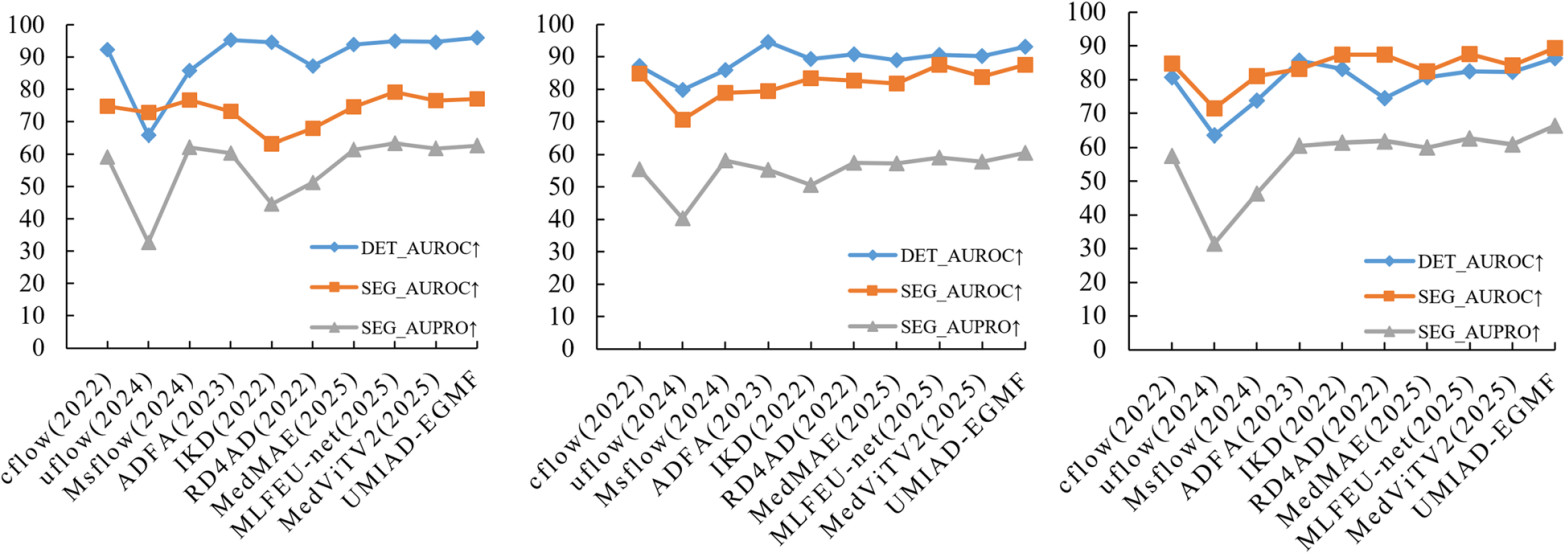
Performance (%) curve comparison of the proposed UMIAD-EGMF with the SOTA methods on the breast ultrasound images (left), brain magnetic resonance imaging (middle), and head computed tomography (right) datasets. The vertical axis represents performance, while the horizontal axis indicates the different methods

Figure 8 presents performance curve comparisons between the proposed UMIAD-EGMF and the SOTA methods on the BUSI, brain MRI, and head CT datasets. These curves clearly show the performance trends of the different methods. It is evident that the proposed UMIAD-EGMF outperformed the SOTA methods in most metrics, which further confirms the superior generalization of the proposed UMIAD-EGMF across different datasets.

**Fig. 9:**
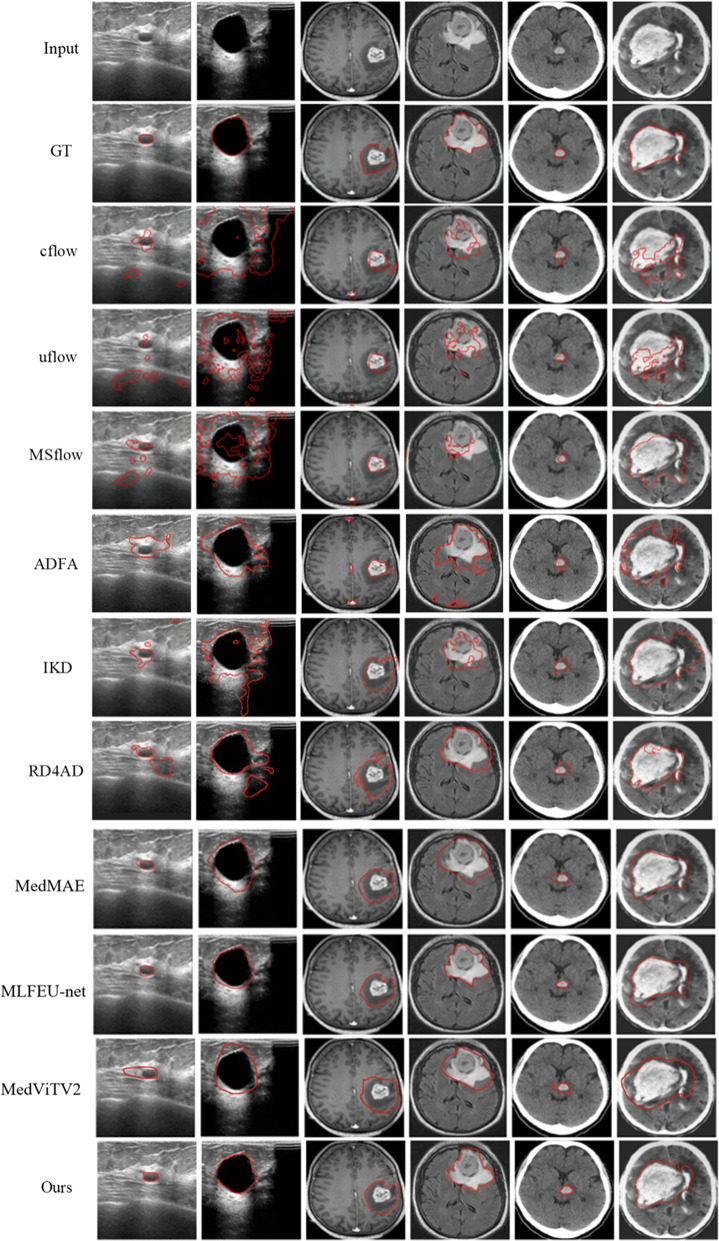
Visualization comparison of detection and segmentation results. The first row shows the original images, the second row shows the ground truth images, and the third to eighth rows show the detection and segmentation results from CFlow [25], UFlow [35], MSFlow [26], ADFA [36], IKD [1], RD4AD [37], MedMAE [27], MLFEU-net [14], and MedViTV2 [15]. The ninth row shows the detection and segmentation results obtained by the proposed UMIAD-EGMF

### Visualization

Figure 9 shows the visual results of the proposed UMIAD-EGMF and SOTA methods across different datasets. The first two columns show example images and their detection and segmentation results on the BUSI dataset, the next two columns show these results on the brain MRI dataset, and the last two columns present those on the head CT dataset. To demonstrate the ability of UMIAD-EGMF to handle multi-scale anomalies, example images containing both small and large anomalies were selected from each dataset. The proposed UMIAD-EGMF exhibited superior accuracy in localizing lesion regions. Specifically, while anomaly detection methods based on the normalizing flow framework tend to produce multiple small detection regions around anomalies, UMIAD-EGMF can eliminate these small interference regions through multi-scale edge information inverse projection. Similarly, while ADFA and other knowledge distillation-based related methods showed relatively few misdetections, UMIAD-EGMF further reduced such misdetections by refining the edge information.

### Performance impact of image resolution

To demonstrate the effect of different input image sizes, experiments were conducted based on varying resolutions of the BUSI, brain MRI, and head CT datasets. The results listed in Table 5 indicate that a 256 $\times$ 256 resolution yielded optimal performance on the BUSI and brain MRI datasets. Conversely, the head CT dataset achieved optimal results with a 128 $\times$ 128 resolution. This difference can be attributed to the positive influence of the higher resolution in capturing critical information for the complex and detailed structures present in the BUSI and brain MRI datasets, while the head CT dataset, which is characterized by larger lesion areas and pronounced gray-scale variations, may sufficiently capture necessary information with a lower resolution of the 128 $\times$ 128. However, the proposed UMIAD-EGMF exhibited the worst performance at the highest resolution of 512 $\times$ 512 in the three datasets, mainly because the noise information increased with the increasing resolution. Therefore, a 256 $\times$ 256 resolution was adopted as the default image size, which balances sufficiently detailed information extraction and complex high-frequency noise suppression.

**Table 5:** Comparative results of different resolutions on the breast ultrasound images, brain magnetic resonance imaging, and head computed tomography datasets (%)

Resolution	DET.AUROC	Seg.AUROC	Seg.AUPRO
**BUSI dataset**			
128 $\times$ 128	88.27	75.24	61.66
256 $\times$ 256	**95.83**	**77.81**	**63.36**
512 $\times$ 512	74.17	73.29	59.20
**Brain MRI dataset**			
128 $\times$ 128	88.12	82.54	59.42
256 $\times$ 256	**90.83**	**86.34**	**59.40**
512 $\times$ 512	82.08	74.50	48.27
**Head CT dataset**			
128 $\times$ 128	80.10	**89.21**	**63.53**
256 $\times$ 256	**82.90**	86.76	62.24
512 $\times$ 512	70.04	84.38	56.40

**Bold** values indicate the best performance (the highest DET.AUROC, Seg.AUROC, or Seg.AUPRO value) for each dataset under different resolutions

### Ablation experiments

To evaluate the effectiveness of the proposed UMIAD-EGMF, related ablation experiments involving different modules, edge extraction methods, and edge aggregation methods were performed on the BUSI, brain MRI, and head CT datasets.

**Table 6:** Comparative results of the ablation experiments on the breast ultrasound images, brain magnetic resonance imaging, and head computed tomography datasets (%)

EGM	EAM	MFF	Dataset	DET.AUROC	Seg.AUROC	Seg.AUPRO
			BUSI	92.43	74.76	59.16
			Brain MRI	87.18	84.84	55.48
			Head CT	80.70	84.77	57.49
			BUSI	93.46	74.99	60.47
✓			Brain MRI	88.71	85.36	57.47
			Head CT	81.15	85.08	58.97
			BUSI	94.16	75.29	62.68
✓	✓		Brain MRI	89.96	86.02	58.37
			Head CT	82.15	85.98	60.70
			BUSI	94.83	74.31	61.48
		✓	Brain MRI	89.03	85.97	57.49
			Head CT	81.81	86.12	59.83
			BUSI	**95.83**	**77.81**	**63.36**
✓	✓	✓	Brain MRI	**90.83**	**86.34**	**59.40**
			Head CT	**82.90**	**86.76**	**62.24**

✓ indicates that the corresponding module (EGM/EAM/MFF) is enabled; **Bold** values indicate the best performance (the highest DET.AUROC, Seg.AUROC, or Seg.AUPRO value) for each dataset in the ablation experiments. *EGM* Edge guidance module, *EAM* Edge aggregation module, *MFF* Multi-scale flow fusion

**Different modules** In Table 6, these results show that the EGM alone can help the network detect anomalies based on edge information. However, the performance improvement of the model was modest. When the EGM and EAM are combined into the proposed model, better performance can be achieved. Additionally, the MFF module can contribute to similar improvements in anomaly detection. Overall, the combination of the three complementary modules achieves the best anomaly detection performance as it integrates key factors, such as edge information, multi-scale flow features, and global contexts, thereby confirming the effectiveness of each module.

**Different edge extraction methods** Image edges are crucial features where significant intensity or color changes occur between different regions, providing vital information about object contours and shapes in image detection and segmentation tasks. To enhance the efficiency of edge extraction in medical images, the proposed EGM was compared with the Prewitt operator and the segment anything model (SAM) [38]. The Prewitt operator detects edges by computing gradient values along the horizontal and vertical directions. SAM is a comprehensive model proposed by ref. [38] and designed for general segmentation tasks in computer vision. From the results listed in Table 7, it is evident that the EGM significantly enhanced the anomaly detection performance, whereas the Prewitt operator and SAM showed inferior performance owing to the complexity and subtlety of medical images.

**Table 7:** Comparative results of the different edge extraction methods on the breast ultrasound image, brain magnetic resonance imaging, and head computed tomography datasets (%)

Method	DET.AUROC	Seg.AUROC	Seg.AUPRO
**BUSI dataset**			
Prewitt	85.54	69.42	56.25
SAM [38]	87.34	72.88	57.05
EGM	**93.46**	**74.99**	**60.47**
**Brain MRI dataset**			
Prewitt	56.56	66.07	41.73
SAM [38]	**92.66**	73.08	54.24
EGM	88.71	**85.36**	**57.47**
**Head CT dataset**			
Prewitt	79.40	78.85	44.67
SAM [38]	79.40	83.82	56.28
EGM	**81.15**	**85.08**	**58.97**

**Bold** values indicate the best performance (the highest DET.AUROC, Seg.AUROC, or Seg.AUPRO value) for each dataset among the compared edge extraction methods

The Prewitt operator offers simplicity and ease of use; however, it is limited by its reliance on local difference extraction, which makes it unable to capture intricate and subtle edge features in medical images. Moreover, its sensitivity to noise often leads to inaccuracies in edge detection results. Similarly, the SAM excels as a pre-trained segmentation model for general images; however, it faces challenges in unsupervised anomaly detection for medical images. Medical images often contain complex and noisy backgrounds outside the training scope of the SAM. Furthermore, in unsupervised learning scenarios, the SAM lacks explicit labels and struggles to effectively detect image edges. In contrast, the EGM is designed for the specific characteristics and demands of medical images, with guidance from prior edge information; therefore, it is better suited for handling the complexities of medical image analysis.

**Different edge aggregation methods** Edge aggregation is a crucial step in processing and integrating edge information, merging and enhancing the extracted edge details to improve segmentation accuracy and robustness. To implement edge information integration, the EAM was introduced to mine low-level appearance and high-level semantic information and obtain aggregated features, thereby enhancing segmentation performance. To demonstrate the effectiveness of the EAM, three different edge feature fusion methods were compared: direct addition (ADD), direct multiplication (MUL), and the EAM. The performance results of these methods are listed in Table 8.

**Table 8:** Comparative results of the different edge aggregation methods on the breast ultrasound image, brain magnetic resonance imaging, and head computed tomography datasets (%)

Method	DET.AUROC	Seg.AUROC	Seg.AUPRO
**BUSI dataset**			
ADD	88.27	75.24	61.66
MUL	87.32	73.75	61.32
EAM	**94.16**	**75.29**	**62.68**
**Brain MRI dataset**			
ADD	88.12	82.54	57.42
MUL	81.62	80.21	56.06
EAM	**89.96**	**86.02**	**58.37**
**Head CT dataset**			
ADD	80.10	89.21	63.53
MUL	76.00	**87.24**	58.78
EAM	**82.15**	85.98	**60.70**

**Bold** values indicate the best performance (the highest DET.AUROC, Seg.AUROC, or Seg.AUPRO value) for each dataset among the compared edge aggregation methods (ADD, MUL, EAM)

The experimental results in Table 8 show that simple fusion methods, such as addition and multiplication, do not fully exploit the edge information, resulting in poor detection and segmentation outcomes. In contrast, the EAM significantly enhanced the anomaly detection performance of the network. Specifically, the addition operation is overly simplistic, merely adding two groups of features pixel-by-pixel, which can lead to overlapping or missing information. The multiplication operation directly multiplies two groups of features pixel-by-pixel, which suppresses some features because multiplication reduces the variability between features. Conversely, EAM is more effective as it mines edge information with greater detail through the attention mechanism and residual connection to significantly improve the segmentation performance.

## Discussion

Based on the comparison experiments and ablation studies, the proposed UMIAD-EGMF successfully handled biomedical image anomaly detection with an excellent performance. By effectively integrating the EGM, EAM, and MFF, the proposed UMIAD-EGMF performed better than other SOTA methods, even though different types of images appeared with complex shapes and varying scales. Most of the existing methods cannot perform accurate anomaly detection, particularly for issues such as blurred edges and varying scales of abnormal regions.

To address these issues, a new unsupervised method for medical image anomaly detection (UMIAD-EGMF) was proposed. UMIAD-EGMF extracts richer edge information with scale adaptability and progressively identifies discriminative information for anomaly detection in medical images. The characteristics of UMIAD-EGMF are as follows: it not only captures contextual information around anomaly boundaries by fusing low-level image features, to enhance the boundary details of abnormal regions via the EGM, but also efficiently integrates the edge information extracted by the EGM into deeper features through the EAM. In addition, UMIAD-EGMF can further merge feature maps at different scales via the MFF module. This allows it to capture common anomaly features, including both subtle and significant multi-dimensional information.

## Conclusions

To address the blurred edges and varying scales of abnormal regions in medical images, this study extended the normalizing flow framework to unsupervised medical image anomaly detection and proposed a novel method (UMIAD-EGMF) tailored for this task. Recognizing the critical role of boundary information in detection and segmentation, a medical image EGM was designed to capture contextual information around anomalous boundaries. This module is integrated into the network to enhance boundary refinement. To effectively fuse structural image features with deep semantic features, an EAM was introduced that combines multi-level features to improve segmentation accuracy. In addition, an MFF module was proposed that exchanges local and global information based on multi-scale pyramid features. This module addresses lesions of varying sizes, thereby enabling the better detection and segmentation of abnormal regions. This study evaluated the proposed UMIAD-EGMF on public medical image anomaly detection datasets, demonstrating improved accuracy and robustness in anomaly detection. However, this study has limitations, as it did not account for common clinical data variations and noise disturbances, such as lighting changes, motion blur, and imaging artifacts. Future work should further model information variation rules using dynamic network architectures to ensure the reliability of the algorithm in the complex environments of real-world clinical applications.

## Data Availability

The datasets used and/or analysed during the current study are available from the corresponding author on reasonable request.

## Abbreviations

UMIAD-EGMF: Unsupervised medical image anomaly detection based on edge guidance and multi-scale flow fusion
CNN: Convolutional neural network
EGM: Edge guidance module
EAM: Edge aggregation module
MFF: Multi-scale flow fusion
AUROC: Area under the receiver operating characteristic curve
AUPRO: Area under the precision-recall curve for object localization
GAN: Generative adversarial network
BUSI: Breast ultrasound images dataset
MRI: Magnetic resonance imaging
SAM: Segment anything model
CAB: Channel attention block
GAP: Global average pooling
MRF: Multi-receptive field
ANN: Artificial eeural network
MP-FocalUNet: Multi-scale parallel focal self-attention U-Net
MAD: Medical anomaly detection
SOTA: State-of-the-art
LoG: Laplacian of Gaussian
MLFEU-net: Multi-scale low-level feature enhancement U-Net
KAN: Kolmogorov-arnold network
CT: Computed tomography
MSFlow: Multi-scale flow-based framework
MedMAE: Medical masked autoencoder
UFlow: U-shaped normalizing flow
ADFA: Attention-augmented differentiable top-k feature adaptation
IKD: Informative knowledge distillation

## References

[1] Cao YK, Wan Q, Shen WM, Gao L (2022) Informative knowledge distillation for image anomaly segmentation. Knowl-Based Syst 248:108846. 10.1016/j.knosys.2022.108846

[2] Zhang Z, Wu CD, Coleman S, Kerr D (2020) DENSE-INception U-net for medical image segmentation. Comput Methods Programs Biomed 192:105395. 10.1016/j.cmpb.2020.105395 32163817 10.1016/j.cmpb.2020.105395

[3] Zeng ZH, Fan CD, Xiao LY, Qu XL (2022) DEA-UNet: a dense-edge-attention UNet architecture for medical image segmentation. J Electron Imaging 31(4):043032. 10.1117/1.JEI.31.4.043032

[4] Kushnure DT, Talbar SN (2022) HFRU-Net: high-level feature fusion and recalibration UNet for automatic liver and tumor segmentation in CT images. Comput Methods Programs Biomed 213:106501. 10.1016/j.cmpb.2021.106501 34752959 10.1016/j.cmpb.2021.106501

[5] Lee C, Liao ZF, Li YZ, Lai QQ, Guo YY, Huang J et al (2023) Placental MRI segmentation based on multi-receptive field and mixed attention separation mechanism. Comput Methods Programs Biomed 242:107699. 10.1016/j.cmpb.2023.107699 37769416 10.1016/j.cmpb.2023.107699

[6] Shao YH, Yuan SL, Zhou XJ, Ye JH (2022) Edge detection algorithm of MRI medical image based on artificial neural network. Procedia Comput Sci 208:136–144. 10.1016/j.procs.2022.10.021

[7] Bakkouri I, Afdel K (2019) Multi-scale CNN based on region proposals for efficient breast abnormality recognition. Multimed Tools Appl 78(10):12939–12960. 10.1007/s11042-018-6267-z

[8] Wang S, Cong Y, Zhu HC, Chen XY, Qu LQ, Fan HJ et al (2021) Multiscale context-guided deep network for automated lesion segmentation with endoscopy images of gastrointestinal tract. IEEE J Biomed Health Inform 25(2):514–525. 10.1109/JBHI.2020.2997760 32750912 10.1109/JBHI.2020.2997760

[9] Chen HY, Li C, Wang G, Li XY, Rahaman MM, Sun HZ et al (2022) GasHis-Transformer: a multi-scale visual transformer approach for gastric histopathological image detection. Pattern Recognit 130:108827. 10.1016/j.patcog.2022.108827

[10] Wang C, Jiang MF, Li Y, Wei B, Li YM, Wang P et al (2025) MP-FocalUNet: multiscale parallel focal self-attention U-Net for medical image segmentation. Comput Methods Programs Biomed 260:108562. 10.1016/j.cmpb.2024.108562 39675195 10.1016/j.cmpb.2024.108562

[11] Dai S, Liu XY, Wei W, Yin XP, Qiao LS, Wang JN et al (2025) A multi-scale, multi-task fusion UNet model for accurate breast tumor segmentation. Comput Methods Programs Biomed 258:108484. 10.1016/j.cmpb.2024.108484 39531807 10.1016/j.cmpb.2024.108484

[12] Zhou T, Zhou Y, He KL, Gong C, Yang J, Fu HZ et al (2023) Cross-level feature aggregation network for polyp segmentation. Pattern Recognit 140:109555. 10.1016/j.patcog.2023.109555

[13] Wang RX, Chen SY, Ji CJ, Fan JP, Li Y (2022) Boundary-aware context neural network for medical image segmentation. Med Image Anal 78:102395. 10.1016/j.media.2022.102395 35231851 10.1016/j.media.2022.102395

[14] Tang RQ, Ning CY (2025) MLFEU-NET: a Multi-scale Low-level Feature Enhancement Unet for breast lesions segmentation in ultrasound images. Biomed Signal Process Control 100:106931. 10.1016/j.bspc.2024.106931

[15] Nejati Manzari O, Asgariandehkordi H, Koleilat T, Xiao Y, Rivaza H (2025) Medical image classification with KAN-integrated transformers and dilated neighborhood attention. Appl Soft Comput 186:114045. 10.1016/j.asoc.2025.114045

[16] Manzari ON, Ahmadabadi H, Kashiani H, Shokouhi SB, Ayatollahi Ahmad (2023) MedViT: a robust vision transformer for generalized medical image classification. Comput Biol Med 157:106791. 10.1016/j.compbiomed.2023.106791 36958234 10.1016/j.compbiomed.2023.106791

[17] Schlegl T, Seeböck P, Waldstein SM, Schmidt-Erfurth U, Langs G (2017) Unsupervised anomaly detection with generative adversarial networks to guide marker discovery. In: Niethammer M, Styner M, Aylward S, Zhu HT, Oguz I, Yap PT et al (eds) Information processing in medical imaging. 25th international conference, Boone, June 2017. Lecture notes in computer science (Image processing, computer vision, pattern recognition, and graphics), vol 10265. Springer, Cham, p 146. 10.1007/978-3-319-59050-9_12

[18] Baur C, Wiestler B, Albarqouni S, Navab N (2019) Deep autoencoding models for unsupervised anomaly segmentation in brain MR images. In: Crimi A, Bakas S, Kuijf H, Keyvan F, Reyes M, van Walsum T (eds) Brainlesion: glioma, multiple sclerosis, stroke and traumatic brain injuries. 4th international workshop, Granada, September 2018. Lecture notes in computer science (Image processing, computer vision, pattern recognition, and graphics), vol 11383. Springer, Cham, p 161. 10.1007/978-3-030-11723-8_16

[19] Schlegl T, Seeböck P, Waldstein SM, Langs G, Schmidt-Erfurth U (2019) f-AnoGAN: Fast unsupervised anomaly detection with generative adversarial networks. Med Image Anal 54:30–44. 10.1016/j.media.2019.01.010 30831356 10.1016/j.media.2019.01.010

[20] Arjovsky M, Chintala S, Bottou L (2017) Wasserstein generative adversarial networks. In: Proceedings of the 34th international conference on machine learning, PMLR, Sydney, 6–11 August 2017. https://dl.acm.org/doi/abs/10.5555/3305381.3305404

[21] Han C, Rundo L, Murao K, Noguchi T, Shimahara Y, Milacski ZÁ et al (2021) MADGAN: unsupervised medical anomaly detection GAN using multiple adjacent brain MRI slice reconstruction. BMC Bioinformatics 22(Suppl 2):31. 10.1186/S12859-020-03936-1 33902457 10.1186/s12859-020-03936-1 PMC8073969

[22] Tian Y, Pang GS, Liu YY, Wang C, Chen YH, Liu FB et al (2024) Unsupervised anomaly detection in medical images with a memory-augmented multi-level cross-attentional masked autoencoder. In: Cao XH, Xu XA, Rekik I, Cui ZM, Ouyang X (eds) Machine learning in medical imaging. 14th international workshop, Vancouver, October 2023. Lecture notes in computer science, vol 14349. Springer, Cham, p 11. 10.1007/978-3-031-45676-3_2

[23] Yu JW, Zheng Y,Wang X, Li W,Wu YS, Zhao R et al (2021) FastFlow: unsupervised anomaly detection and localization via 2D normalizing flows. arXiv preprint arXiv: 2111.07677. 10.48550/arXiv.2111.07677

[24] Rudolph M, Wandt B, Rosenhahn B (2021) Same same but DifferNet: semisupervised defect detection with normalizing flows. In: Proceedings of the IEEE winter conference on applications of computer vision, IEEE, Waikoloa, 3–8 January 2021. 10.1109/WACV48630.2021.001957

[25] Gudovskiy D, Ishizaka S, Kozuka K (2022) CFLOW-AD: real-time unsupervised anomaly detection with localization via conditional normalizing flows. In: Proceedings of the IEEE/CVF winter conference on applications of computer vision, IEEE, Waikoloa, 3–8 January 2022. 10.1109/WACV51458.2022.00188

[26] Zhou YX, Xu X, Song JK, Shen FM, Shen HT (2025) MSFlow: multiscale flowbased framework for unsupervised anomaly detection. IEEE Trans Neural Netw Learn Syst 36(2):2437–2450. 10.1109/TNNLS.2023.3344118 10.1109/TNNLS.2023.3344118 38194384

[27] Gupta A, Osman I, Shehata MS, Braun WJ, Feldman RE (2025) MedMAE: a self-supervised backbone for medical imaging tasks. Computation 13(4):88. 10.3390/computation13040088

[28] He KM, Zhang XY, Ren SQ, Sun J (2016) Deep residual learning for image recognition. In: Proceedings of the IEEE conference on computer vision and pattern recognition, IEEE, Las Vegas, 27–30 June 2016. 10.1109/CVPR.2016.90

[29] Woo S, Park J, Lee JY, Kweon IS (2018) CBAM: convolutional block attention module. In: Ferrari V, Hebert M, Sminchisescu C, Weiss Y (eds) Computer vision – ECCV 2018. 15th European conference, Munich, September 2018. Lecture notes in computer science (Image processing, computer vision, pattern recognition, and graphics), vol 11211. Springer, Cham, p 3. 10.1007/978-3-030-01234-2_1

[30] Rezende DJ, Mohamed S (2015) Variational inference with normalizing flows. In: Proceedings of the 32nd international conference on machine learning, JMLR.org, Lille, 6–11 July 2015. https://proceedings.mlr.press/v37/rezende15

[31] Al-Dhabyani W, Gomaa M, Khaled H, Fahmy A (2020) Dataset of breast ultrasound images. Data Brief 28:104863. 10.1016/j.dib.2019.10486 10.1016/j.dib.2019.104863 PMC6906728 31867417

[32] Menze BH, Jakab A, Bauer S, Kalpathy-Cramer J, Farahani K, Kirby J et al (2015) The multimodal brain tumor image segmentation benchmark (BRATS). IEEE Trans Med Imaging 34(10):1993–2024. 10.1109/TMI.2014.2377694 25494501 10.1109/TMI.2014.2377694 PMC4833122

[33] FelipeKitamura (2018) Head CT-hemorrhage. 10.34740/kaggle/dsv/152137. Accessed 13 Jan 2023

[34] Ardizzone L, Kruse J, Wirkert S, Rahner D, Pellegrini EW, Klessen RS et al (2018) Analyzing inverse problems with invertible neural networks. arXiv preprint arXiv: 1808.04730. 10.48550/arXiv.1808.04730

[35] Tailanian M, Pardo Á, Musé P (2024) U-flow: a U-shaped normalizing flow for anomaly detection with unsupervised threshold. J Math Imaging Vis 66(4):678–696. 10.1007/s10851-024-01193-y

[36] Huang YM, Liu GL, Luo YR, Yang G (2023) ADFA: attention-augmented differentiable Top-K feature adaptation for unsupervised medical anomaly detection. In: Proceedings of the 2023 IEEE international conference on image processing (ICIP), IEEE, Kuala Lumpur, 8–11 October 2023. 10.1109/ICIP49359.2023.10222528

[37] Deng HQ, Li XY (2022) Anomaly detection via reverse distillation from one-class embedding. In: Proceedings of the IEEE/CVF conference on computer vision and pattern recognition, IEEE, New Orleans, 18–24 June 2022. 10.1109/CVPR52688.2022.00951

[38] Kirillov A, Mintun E, Ravi N, Mao HZ, Rolland C, Gustafson L et al (2023) Segment anything. In: Proceedings of the IEEE/CVF international conference on computer vision, IEEE, Paris, 1–6 October 2023. 10.1109/ICCV51070.2023.00371

